# Stimulation of Nucleotide Oligomerization Domain and Toll-Like Receptors 2 to Enhance the Effect of Bacillus Calmette Guerin Immunization for Prevention of Mycobacterium Tuberculosis Infection: Protocol for a Series of Preclinical Randomized Controlled Trials

**DOI:** 10.2196/13045

**Published:** 2019-06-08

**Authors:** Michael Eisenhut

**Affiliations:** 1 Luton&Dunstable University Hospital NHS Foundation Trust Luton United Kingdom

**Keywords:** innate immunity, BCG, bacillus calmette guerin vaccine, Mycobacterium tuberculosis infection, toll-like receptors

## Abstract

**Background:**

Bacillus calmette guerin (BCG) immunization has been associated with a reduction in *Mycobacterium tuberculosis* (MTB) infection. BCG immunization has been shown to enhance innate immunity. This effect of BCG can be explained by an enhancing effect on innate immunity.

**Objective:**

This study aimed to test the following hypotheses: (1) BCG immunization can prevent infection with MTB, (2) prevention of infection occurs via stimulation of NOD2 (nucleotide oligomerization domain) and toll-like receptors 2 (TLR2), and (3) the effect of BCG immunization on prevention of infection with MTB can be enhanced by giving stimulators of NOD2 and TLR2.

**Methods:**

To detect the influence of immunization on infection rates, the ultralow dose (ULD) infection model is used. The infection rate of mice vaccinated with BCG and exposed after 6 weeks to ULD of MTB and unvaccinated mice are compared via cultures of lung homogenates and interferon (IFN) gamma release assay. If a reduced infection rate by BCG immunization is confirmed, the experiment is repeated by giving BCG combined simultaneously or in time sequence with the enhancers of innate immunity murabutide or beta-glycan. The influence of murabutide or beta-glycan alone on infection rates is investigated. To quantify the contribution of innate immunity levels of tumor necrosis factor, IFN gamma expression, histone H3 K4me3 trimethylation, and concentrations of monocytes with features of activation of innate immunity as defined by the Ly6Chigh as well as CD11b positive phenotype in immunized versus unimmunized infected and uninfected mice in the various immunization protocols is compared. The experiments will be repeated with prior application of the inhibitors of epigenetic programming of innate immunity histone methyltransferase inhibitor 5’-deoxy-5’-methylthio-adenosine and histone acetyl transferase inhibitor epigallocatechin-3-gallate. The influence of BCG on innate immunity is further corroborated by a prospective observational study in human infants.

**Results:**

Investigations of derivatives of muramyl dipeptide (MDP) to enhance early immunity in the C57BL/6 mouse strain (mice aged 7 weeks) by another group used 300 micrograms per mouse of oil-associated 6-0-mycoloyl-N-acetylmuramyl-L-alanyl-D-isoglutamine (mycol-MDP) 50/50 mixed with Freund’s incomplete adjuvant. Comparison of colony-forming unit (CFU) count in the lungs 3 weeks after aerosol challenge with *Mycobacterium bovis* of groups (n=5) between groups receiving mycol-MDP in oil emulsion (see above) versus controls (n=5) showed a significantly lower CFU count of 94.5 x106 (SD 22.0) in cases versus controls with 204.0 X 106 (SD 77.6). It is important to note that after elimination of T-cells in this model, a reduction of CFU in lungs of mice treated with mycol-MDP persisted albeit without statistical significance, which was possibly related to the small number of animals used.

**Conclusions:**

Demonstration of a reduction of MTB infection by enhancement of innate immunity could show a new approach to improving vaccine efficacy against this pathogen.

**International Registered Report Identifier (IRRID):**

PRR1-10.2196/13045

## Introduction

### Background

Bacillus calmette guerin (BCG) immunization has been associated with a reduced reactivity of interferon (IFN) gamma release assays for Mycobacterium tuberculosis (MTB) infection in exposed children [1]. This finding is compatible with a reduction of infection because of an abortion of infection before engagement of T-effector cells induced by an enhancement of innate immunity by BCG immunization [2,3]. Previous vaccination studies used mouse models with infection of all mice and vaccine efficacy identified by reduced organ colony forming units. To demonstrate that BCG immunization can reduce MTB infection experimentally, one needs to reduce the aerosol inoculation dose to an amount that does not overwhelm first line innate immunity and which enables detection of a lack of engagement of adaptive immunity in form of T-effector cells. In the ultralow dose (ULD) model, infection rate is measurable, and the ULD model can be used to identify host or bacterial factors that modulate the initial infection event and may be used to assess the effects of vaccination on the infection event by comparing infection rates in control and vaccinated groups exposed to the same aerosol [4]. Should a reduction of MTB infection in BCG immunized mice be confirmed with the above experiment, further experiments can be conducted investigating both the underlying mechanisms as well as ways to enhance the effect of BCG by identification of compounds able to reduce MTB infection by enhancing innate immunity with and without concomitant BCG immunization. Components of innate immunity potentially modifiable are pattern recognition receptor signaling including toll-like receptor 2 (TLR2) signaling by action of muramyl dipeptide (MDP) derivatives with a single octanoyl or stearoyl fatty acid chain, nucleotide oligomerization domain (NOD) 1 by H-Ala-D-γ-Glu-diaminopimelic acid, and NOD 2 signaling also by use of MDP, which is a stimulator of NOD expression and possibly murabutide (see rationale and protocol in [3]), a nontoxic derivative of MDP [5-10]. Simultaneous stimulation of TLR 2 and NOD 2 has hereby been shown to be associated with a synergistic effect on antimycobacterial cytokine production [6]. The most powerful stimulator of innate immunity may be yeast-derived beta glucan through stimulation of the intramembranous C-type lectin receptor dectin-1 [11,12]. Essential effector mechanism of innate immunity coupled to pattern recognition receptor stimulation is the induction of tumor necrosis factor (TNF) and IFN gamma production in monocytes through action of nuclear factor kappa B. TNF and IFN gamma enhance autophagy and production of nitric oxide metabolites toxic to mycobacteria [3,13].

NOD 2 stimulation has previously been shown to lead to recruitment of Ly6C^high^ and CD11b positive monocytes, which were found to be associated with NOD2 stimulation, and which can, therefore, be used as a marker for stimulation of innate immunity [14].

### Hypotheses

The hypotheses of this research are as follows:

- BCG immunization can prevent infection with MTB
- Prevention of infection occurs via stimulation of NOD 2 and TLR2
- The effect of BCG immunization on prevention of infection with MTB can be enhanced by giving stimulators of NOD 2 and TLR2

### Explanation of the Hypothesis

The reduction of MTB infection found in BCG immunized children exposed to MTB can be explained by stimulation of innate immunity, which is mediated among other pathways through stimulation of NOD2 and TLR2. Stimulation of receptors mediating innate immunity in addition to stimulation by BCG could, therefore, enhance the effect of BCG on reduction of MTB infection.

### Testing the Hypothesis

#### Objectives

The objectives of this research are as follows:

1. To confirm the validity of a mouse model of a partial infection with MTB.
2. To investigate whether BCG immunization can reduce infection with MTB.
3. To investigate whether stimulators of NOD2, TLR2, or dectin-1 receptors can enhance an effect of BCG on reduction of infection with MTB.
4. To investigate the influence of T-suppressor cells on a reduced reactivity in the gamma IFN release assay results in MTB exposed BCG vaccinated mice.
5. To investigate whether a reduction of infection with MTB in BCG-immunized mice and in mice injected with enhancers of innate immunity is associated with a significant increase in markers of enhancement of innate immunity such as H3K4 trimethylation of monocyte deoxyribonucleic acid (DNA) and increase in Ly6C^high^ and CD11b positive monocytes in uninfected mice.
6. To investigate whether a reduction of infection with MTB is associated with an increased intracellular expression of TNF and IFN gamma messenger ribonucleic acid (mRNA) levels in monocytes in uninfected mice.
7. To establish whether inhibition of epigenetic programming can reduce the effect of BCG immunization and stimulators of innate immunity on MTB infection and markers of activation of innate immunity.

## Methods

### Definitions

MTB infection in the mouse model is defined as MTB positive culture of lung homogenates or positive IFN gamma release assay performed in mice with culture negative lungs after sacrificing at 18 weeks after exposure to an aerosol of MTB.

### The Infection Model

To detect the influence of immunization on infection rates, the ULD infection model is used [4]: Groups of female adult C57BL/6 mice are exposed to ULD MTB aerosol for 20 min. An adult mouse model is used because the effect of BCG immunization on MTB has also been detected in adult humans [15,16], and tissue (spleen) required for IFN gamma release assay in adult mice will be big enough to detect specific reactive T-effector cells and provide the cell populations for subsequent experiments. Before using large numbers of mice (a total of 222 initially) for the first experiment, the reproducibility of the ULD model is tested by initially exposing 28 mice (the number is identical to the number of the first reported ULD experiment in mice in Saini et al (2012) [4] with at least 10 mice expected to have detectable evidence of infection. The number of mice with a detectable infection is expected to be at least as large because the definition of infection in our study, unlike Saini et al [4] who only used lung cultures to confirm infection, includes enzyme-linked immunospot (ELISpot) positivity in culture negative mice.

### Sample Size Calculation

With an ULD MTB aerosol model, it is expected that with a starting inoculum of OD 2.5x 10^-4^ with a prenebulizer bacterial count of 3.05 x10^4^ colony-forming unit (CFU)/ml, the result is 1.1 CFU presented/ per mouse with a predicted infection rate of 36% (36/100) [4]. BCG immunization was found to be associated with a reduction of the infection rate from 67/143 (47%) to 16/56 (29%) in children in an outbreak investigation in the United Kingdom [17]. This study was chosen for the sample size calculation because the geographical setting of the United Kingdom is more applicable to the laboratory setting with its reduced likelihood of infection with nontuberculous mycobacteria, absence of malnutrition, and lack of concomitant helminth infections, which can all influence both effectiveness of BCG immunization as well as the results of IFN gamma release assays by induction of bias away from a Th-1 response essential for antimycobacterial immunity. For the comparison of the BCG immunized and nonimmunized groups of mice using the ULD model to detect a difference of 18% in prevalence of infection between groups with 80% power at a significance level of 5%, 111 mice need to be used in each group.

### The Composition of Experimental Groups

The composition of the experimental groups is as follows:

1. Control group: Mice exposed to ULD of MTB and not vaccinated.
2. Intervention group: Mice vaccinated with BCG at a dose of 5x10^4^ CFU per mouse and exposed after 6 weeks to ULD of MTB.

### Randomization and Blinding Procedures

The mice are randomized into each group after labeling with a number (attached to the tail) using computer randomization [18]. The numbers in each group are registered and allocation concealed from members processing mice and analyzing data. The persons processing the mice for mycobacterial culture and IFN gamma release assay as well as involved in culture and assay procedure are blinded to the allocation of the numbers to groups. The persons analyzing the data are blinded to which group received the immunization.

### Care and Use of Laboratory Animals and Ethics

All animal work was carried out in accordance with the UK Animal (Scientific Procedures) Act 1986, under appropriate Personal and Project licenses. The study will only go ahead after approval by the Institutional Animal Use Ethics Committee. Animals will be housed in appropriate biological containment facilities according to the code of practice for the housing and care of animals bred, supplied, or used for scientific purposes as outlined in [19].

### Outcomes

The outcomes are as follows:

1. Proportion of exposed mice found not to be infected with MTB: This is taken as confirmation of the validity of the model of partial infection with MTB in mice.
2. Reduction of the infection rate in BCG immunized mice compared with controls. This is taken as confirmation that BCG immunization can reduce the infection event itself.
3. Infection rate with MTB after application of enhancers of innate immunity.

### Detection of *Mycobacterium tuberculosis* on Culture in Infected Mice

Lungs of mice succumbing before 18 weeks after exposure to aerosol and lungs of mice sacrificed at 18 weeks because alive at that time after exposure to aerosol are put in 0.9% sodium chloride and sent to a collaborating microbiological laboratory for culture.

### Detection of *Mycobacterium tuberculosis* Infection by Interferon Gamma Release Assay

IFN gamma release assays are conducted in all mice surviving to 18 weeks in the form of an ELISpot assay using spleen cells.

### Enzyme-Linked Immunospot

The procedure below was taken in modified form from a published protocol [20]:

1. Preparation of ELISpot 96-well plate by coating with capture anti-IFN-gamma antibody:
  - Pretreatment of plates with 200 microl/well of 70% ethanol for 10 min.
  - Rinsing the wells with 200 microlitles/well of tissue culture medium in PBS 3 times (5 min each wash).
  - Coating of plates with 100 microl/well of 10 microl/ml solution of capture, rat antimouse IFN-gamma antibody (clone R4-6A2) in 1 X PBS, and incubation overnight at 4 degrees Celsius.
2. The spleen of mice sacrificed after survival at 18 weeks is removed and put in RPMI-1640 medium supplemented with 100 IU ml^−1^ penicillin, 50 µg ml^−1^ streptomycin, 1 mM l-glutamine, 25 mM HEPES, 1 mM sodium pyruvate, 5 × 10^−5^ M β-mercaptoethanol, vitamins and nonessential amino acids (Gibco-Invitrogen), and 10% endotoxin-tested heat-inactivated fetal bovine serum (Atlas Biologicals) as described previously [21].
3. The spleen is digested with an enzyme mixture containing 1 mg ml^−1^ collagenase type IV (Sigma-Aldrich) and 25 U ml^−1^ DNase (Roche) in supplemented RPMI-1640 at 37 °C for 1 h. The digested spleen is pressed through a 70-µm pore size cell strainer (BD Falcon) to obtain a single cell suspension. The erythrocytes are lysed with RBC lysis buffer (eBioscience) at 22°C, and cells are washed extensively (×4), resuspended in supplemented RPMI-1640, and counted using an automated cell counter (Countess, Invitrogen) employing the trypan blue dye exclusion method. Cell concentration is adjusted to 10^6^ cells ml^−1^ in supplemented RPMI-1640 before addition to appropriate wells.
4. All reagents are brought to room temperature, except the detection antibody concentrate and dilution buffer, which should remain at 2 to 8°C. All samples and controls are assayed in duplicate. An assay record template is used to record controls and samples assayed. All wells in the microplate are filled with 200 μL of sterile culture media and incubated for approximately 20 min at room temperature. When cells are ready to be plated, the culture media is aspirated from the wells.
5. 100 μL of the appropriate cells are added to each well. The cells are plated in duplicate with 10^5^ cells per well, incubated (37 degrees, 5% CO_2_ ) 24 hours with media, 4 micrograms/ml Con A, 2.5 micrograms/ml anti-CD3 (clone 145-2C11), 2 micrograms/ml MTB Erdman CFP, and 10 micromol of an ESAT-6 MHC class II-restricted epitope peptide (MTEQQWNFAGIEAAA. Cells are incubated in a humidified 37 °C CO2 incubator. The controls are:
  - Positive control 1 to check T-effector cells for ability to release IFN gamma controls: Cells stimulated with phytohaemagglutinin are added to 2 wells.
  - Positive control 2 to check binding capacity of coating antimouse IFN gamma antibodies: recombinant mouse IFN-γ is added to 2 wells.
  - Negative control 1: Unstimulated Control cells—using the same number of unstimulated cells as stimulated cells.
  - Negative control 2: Instead of cells only sterile culture media is used.
  - Negative control 3: Detection antibody control: Phosphate buffered saline is substituted for the detection antibody.
6. Each well is aspirated and washed and the process repeated 3 times for a total of 4 washes. Wash is performed by filling each well with wash buffer (250-300 μL) using a squirt bottle, manifold dispenser, or auto-washer. Complete removal of liquid at each step is essential to good performance. After the last washing step, any remaining wash buffer is removed by aspirating or decanting. The plate is inverted and blotted against clean paper towels.
7. 100 μL of diluted detection antibody mixture is added into each well and incubated overnight at 2 to 8°C.
8. The washing procedure is repeated as described in step 7.
9. 100 μL of diluted Streptavidin-AP is added into each well and incubated for 2 hours at room temperature.
10. The wash procedure is repeated as described in step 7.
11. 100 μL of BCIP/NBT Chromogen is added into each well and incubated for 1 hour at room temperature. The plates are protected from light.
12. The chromogen solution is discarded from the microplate, and the microplate rinsed with deionized or distilled water. The microplate is inverted and tapped to remove excess water. The flexible plastic under drain is removed from the bottom of the microplate, the bottom of the plate wiped thoroughly with paper towels, and dried completely either at room temperature (60-90 min) or 37°C (15-30 min).
13. Spot forming is counted manually with a dissecting microscope. More than 6 spots constitute a positive result indicating previous infection with MTB.

### Data Analysis

Percentage of infected mice between groups is compared by chi-square or Fisher exact testing as appropriate. Statistical significance set to be indicated by a *P* value of <.05.

### Experiments to Identify Compounds Associated with an Increased Protection Against *Mycobacterium tuberculosis* Infection and Identification of Mechanisms Related to Protection Against Infection

#### Sample Size Calculation

A sample size calculation in the experiments below is guided by the outcome of the experiments conducted above.

#### Randomization and Blinding Procedures

See above.

#### The Composition of Experimental Groups

The composition of the experimental groups is as follows:

1. Negative control group: Mice exposed to ULD of MTB and not vaccinated.
2. Positive control: Mice vaccinated with BCG a dose of 5x10^4^ CFU per mouse and exposed after 6 weeks to ULD of MTB.
3. Mice vaccinated with BCG followed by inoculation with murabutide at a dose of 0.1mg/kg 2 months later followed after 6 weeks by exposure to ULD of MTB aerosol.
4. Mice vaccinated with BCG followed by inoculation with beta-glucan at a dose of 0.1mg/kg 2 months later followed after 6 weeks by exposure to ULD of MTB aerosol.
5. Mice vaccinated with BCG and simultaneously with murabutide mixed in the same syringe at a dose of 0.1mg/kg and exposed after 6 weeks to ULD of MTB.
6. Mice vaccinated with BCG and simultaneously with beta-glucan mixed in the same syringe at a dose of 0.1mg/kg and exposed after 6 weeks to ULD of MTB.
7. Mice vaccinated with murabutide at a dose of 0.1mg/kg and exposed after 6 weeks to ULD of MTB.
8. Mice vaccinated with beta-glucan at a dose of 0.1mg/kg and exposed after 6 weeks to ULD of MTB.

#### Detection of *Mycobacterium tuberculosis* on Culture in Infected Mice

Lungs of mice succumbing before 18 weeks after exposure to aerosol and lungs of mice sacrificed at 18 weeks because alive at that time after exposure to aerosol are put in 0.9% sodium chloride and sent to a collaborating microbiological laboratory for culture.

#### Detection of *Mycobacterium tuberculosis* Infection by Interferon Gamma Release Assay

IFN gamma release assays are conducted in all mice surviving to 18 weeks in the form of an ELISpot assay using spleen cells.

#### Data Analysis

Percentage of infected mice between groups is compared by chi-square or Fisher exact testing as appropriate. Statistical significance set to be indicated by a *P* value of <.05.

### Investigation of the Role of Epigenetic Programming of Innate Immunity in Protection Against *Mycobacterium tuberculosis* Infection

The following two courses of investigation will be undertaken:

- To assess whether clearance of infection in BCG-immunized mice is related to activation of innate immunity, the influence of inhibition of epigenetic programming on MTB infection is examined
- To investigate whether protection from infection is related to activation of innate immunity H3K4 trimethylation of monocyte DNA and numbers of activated monocytes and degree of intracelluar TNF and IFN gamma, mRNA expression is measured and degree of methylation and numbers of activated monocytes and degree of TNF and IFN gamma expression compared between infected and uninfected mice

### Experimental Procedure

Above experiment is conducted with the modification that mice receiving BCG, murabutide or beta glucan alone, or murabutide or beta glucan and BCG are at the time of the first injection of these agents intraperitoneally injected with inhibitors of training of innate immunity the histone methyltransferase inhibitor 5’-deoxy-5’-methylthio-adenosine, and the histone acetyl transferase inhibitor epigallocatechin-3-gallate [5,11] to abolish the training effect on innate immunity and infection rates compared with controls receiving these agents without prior injection with inhibitors of epigenetic programming.

In mice in all groups at death or at sacrifice at 18 weeks after aerosol exposure with MTB, spleen cells are used for measurement of H3K4 trimethylation, enumeration of activated monocytes by flow cytometry, and measurement of intracellular TNF and IFN gamma expression.

### Details of Methods

#### Determination of Epigenetic Programming in Form of Histone Modification by Chromatin Immuno Precipitation

Chromatin immunoprecipitation (ChIP) is a technique allowing the analysis of the histone modifications associated with specific genomic regions in the context of intact cells. ChIP is then used to connect epigenetic marks to intergenic regions, active coding regions, and/or silenced coding regions.

The main steps of the ChIP technique are cell fixation, chromatin shearing, immunoselection, immunoprecipitation (IP), and analysis of the immune-precipitated (IP’d) DNA.

In short, cells are briefly fixed with a reversible cross-linking agent. Next, the cross-linked chromatin (DNA-protein) is sheared, and the DNA fragments associated with the protein of interest are immunoprecipitated using specific antibodies. Finally, the IP’d DNA is examined for the presence of particular sequences by quantitative polymerase chain reaction (qPCR). Enrichment of specific sequences in the precipitate indicates that the sequences are associated with the protein of interest in vivo.

### Protocol for Chromatin Immunoprecipitation

The following protocol is taken from the Instruction Manual Version 2 01.14 available from a source quoted below [22]**:**

1. Cell fixation and collection of 10x10^6^ cells splenic cells obtained for the ELISpot procedure.
2. Cell lysis and chromatin shearing.
3. Immuno-selection and precipitation using antibody antihistone H3 K4me3.
4. DNA purification.
5. qPCR of TNF and IFN gamma genes.
6. Determination of occupancy of the 2 promoters by the modified histones is evident based on fluorescent qPCR analysis of immunoprecipitated DNA.

Quantification of intracellular cytokine mRNA expression:

1. Cells are harvested by trypsinization, and total RNA extracted using TRIzol reagent (Gibco BRL, Gaithersburg, MD). A total of 5 μg of total RNA is reverse-transcribed according to the manufacturer’s protocol (Superscript II Preamplification System, Gibco BRL) using oligo (dT) as primer.
2. cDNA prepared from 0.5 μg RNA is subjected to PCR using murine gene-specific primers for TNF and IFN gamma. All of the primer pairs span at least 1 intron in the corresponding genomic DNA. Positive RNA controls are performed previously to confirm specificity of primer pairs. Negative controls are performed by omitting the RT step or the cDNA template from PCR amplification.
3. For semiquantitative PCR, target sequences for TNF alpha and IFN gamma are amplified at 56°C between 22 and 32 cycles to yield visible products within the linear amplification range.
4. PCR products are separated by electrophoresis on a 2% agarose gel and stained with ethidium bromide.
5. All reverse transcription PCR bands at the expected size are also directly sequenced to confirm their identity.
6. Glyceraldehyde-3-phosphate dehydrogenase or acidic ribosomal phosphoprotein are used as internal controls.

### Investigation of the Change in the Monocyte Phenotype as Aarker of Nucleotide Oligomerization Domain 2 and Beta Glucan–Induced Activation

As a correlate of NOD2 activation by MDP derivatives flow cytometry on spleen cells to detect Ly6C^high^ as well as CD11b positive monocytes is performed, and numbers of these monocyte populations is compared between immunized and not immunized mice in each group.

#### Outcomes

The outcomes are as follows:

- MTB infection defined as positive culture of lung homogenates in mice with active tuberculosis or positive IFN gamma release assay performed in spleen cells of mice with culture negative lungs after sacrificing at 18 weeks after aerosol challenge
- Level of TNF and IFN gamma and histone H3 K4me3 trimethylation in infected versus uninfected mice
- Concentrations of monocytes with features of activation of innate immunity as defined by Ly6C^high^ as well as CD11b positive phenotype
- Change in TNF and IFN gamma mRNA expression in infected versus uninfected mice
- Correlation of TNF and IFN gamma mRNA expression with monocyte phenotype and levels of histone H3 K4me3 trimethylation

### Testing the Role of T-Suppressor Cells in Apparent Reduction of Infection Rate as Measured by Interferon Gamma Release Assay

The experiment is repeated with all groups of mice except 1 control group undergoing depletion of T-suppressor cells by injection of the monoclonal antibody 2.43, which reacts against an antigen present on T suppressor/cytotoxic (CD8) cells before injection of murabutide, BCG or murabutide and BCG as described previously [23].

For data analysis, the rate of infection as measured by ELISpot assay is compared between BCG immunized and/or murabutide injected groups.

## Results

### Evidence Supporting the Hypothesis

The first experiments using derivatives of MDP to enhance early immunity in the C57BL/6 mouse strain (mice aged 7 weeks) used 300 micrograms per mouse of oil-associated 6-0-mycoloyl-N-acetylmuramyl-L-alanyl-D-isoglutamine (mycol-MDP) 50/50 mixed with Freund’s incomplete adjuvant suspended with 0.9% sodium chloride solution with 0.2% Tween aiming at a final oil concentration of 3% and given intravenously. Comparison of CFU count in the lungs 3 weeks after aerosol challenge with *Mycobacterium bovis* of groups (n=5) between groups receiving mycol-MDP in oil emulsion (see above) versus controls (n=5) receiving only oil emulsion showed a significantly lower CFU count of 94.5 x10^6^ (SD 22.0) in cases versus controls with 204.0 X 10^6^ (SD 77.6) [24]. It is important to note that after elimination of T-cells in this model (by irradiation and thymectomy), a reduction of CFU in lungs of mice treated with mycol-MDP persisted albeit without statistical significance, which was possibly related to the small number of animals used. This result confirmed the findings of a previous study of the same group in the mouse strain C3H/He [25].

The BCG primed increased TNF release by monocytes has been shown to be related to effects of epigenetic programming in form of stimulation of trimethylation of histone H3 at lysine 4 (H3K4). Establishment of innate immunity in monocytes could hereby be inhibited by use of inhibitors of epigenetic programming [8].

### Evidence Against the Hypothesis

The most important alternative hypothesis, which could be advanced to explain the apparently reduced infection rate on gamma IFN release assay testing in BCG immunized humans or mice, is clonal imprinting (previously termed *original antigenic sin* phenomenon) where previous exposure to an antigen (in this case BCG) leads to reinforcement of the immune reaction to this antigen on exposure of the immune system to a similar antigen (MTB) containing this previous antigen (in this case BCG) rather than a reaction to the new antigen (MTB-specific epitopes) not contained in the antigen mixture of the previous exposure. This process has been found to be dependent on the action of the cytokine IL-10 [26]. IL-10 is hereby produced by nonantigen specific T-suppressor cells [27], thus postulated to reduce IFN gamma release in MTB exposed individuals in the gamma IFN release assays if there was a previous exposure to BCG. This alternative hypothesis can be tested by elimination of T-suppressor cells.

The protective effect of BCG immunization against infection with MTB is reduced in low income countries [1], but its effect against other infections, which is more consistent with enhancement of innate immunity, is considerable [28]. This is more supportive of the hypothesis that the reduction of infection with MTB is also because of T-effector cells, which can be influenced by malnutrition and helminth infection triggered regulatory T-cell activation and not because of effects of BCG on innate immunity. The conclusion would, therefore, be that innate immunity can actually not be enhanced by antigens similar to BCG derived substances, which can stimulate NOD2 or TLR2.

## Discussion

### Impact of Confirmation of Reduction of *Mycobacterium tuberculosis* Infection by Enhancement of Innate Immunity

Should above experiments confirm that a reduction in MTB infection by BCG immunization can be confirmed in the mouse model and enhanced by amplifiers of innate immunity, this approach would have to be then tested in nonhuman primates and if again successful would lead to an enhanced BCG immunization schedule in humans. The potential of enhancing innate immunity extends far beyond immunization against MTB infection, and one may want to investigate the impact of such an approach on infections with all other pathogens.

### Testing Bacillus Calmette Guerin Enhancement of Innate Immunity in Humans

#### The Influence of Neonatal Bacillus Calmette Guerin Immunization on Neutrophil-Mediated Innate Immunity in Infants at Risk of HIV Infection

BCG immunization is known to influence innate immunity against MTB infection [8] associated with its effect on cells of the monocyte/macrophage lineage. Neutrophil leucocytes may be an important contributor to innate immunity against MTB infection in collaboration with macrophages [29]. Infants of mothers with human immunodeficiency virus (HIV) infection and undetectable HIV viral load at 36 weeks gestation have routinely blood samples taken at birth and at 6 weeks of age to check for evidence of HIV infection, and a BCG immunization is given after birth. In infants born to mothers with detectable viral load at 36 weeks gestation, BCG immunization is withheld until after HIV DNA PCR is negative at 3 months of age. From this information one can formulate the following hypothesis: BCG immunization increases neutrophil-mediated innate immunity.

Outcomes suitable for investigation of an effect of BCG immunisation on neutrophil innate immunity are: change in neutrophil associated antimycobacterial activity in whole blood associated with BCG immunization as measured using MTB luciferase assay adapted to small sample sizes [30].

Neutrophil leucocyte attributable innate immunity to MTB could be compared in infants with and without BCG immunization born to mothers with HIV infection.

To assess neutrophil leucocyte attributable innate immunity, 1 ml of blood is taken in addition to the routine bloods taken: 0.5 ml subjected to MTB luciferase assay (30) and 0.5 to MTB luciferase assay after neutrophil depletion. The same is repeated at 6 weeks of age (after the BCG immunization in the immunized group). Groups with and without BCG immunization are compared with control for general changes of innate immune response in the postnatal period.

#### Impact

In vitro studies could determine the component of BCG, which enhances neutrophil antimycobacterial activity by exposure of neutrophils in vitro to components of BCG and measuring antimycobacterial activity by MTB luciferase assay before and after exposure to each component; starting with muramyl dipeptide, a compound produced by BCG previously associated with enhancement of antimycobacterial activity in monocytes [1].

Should an increase of neutrophil dependent innate immunity by BCG immunization be confirmed, a change in neutrophil-mediated immunity could serve as an outcome measure in future trials of BCG immunization and correlated with negative IFN gamma release assay results in exposed people in future vaccine trials.

## Abbreviations

BCG: bacillus calmette guerin
CFU: colony-forming unit
ChIP: chromatin immunoprecipitation
DNA: deoxyribonucleic acid
ELISpot: enzyme-linked immunospot
IFN: interferon
IP: immunoprecipitation
MDP: muramyl dipeptide
mRNA: messenger ribonucleic acid
MTB: *Mycobacterium tuberculosis*
NOD: nucleotide oligomerization domain
qPCR: quantitative polymerase chain reaction
TLR2: toll-like receptors 2
TNF: tumor necrosis factor
ULD: ultralow dose
